# Twelve Weeks of Sprint Interval Training Improves Indices of Cardiometabolic Health Similar to Traditional Endurance Training despite a Five-Fold Lower Exercise Volume and Time Commitment

**DOI:** 10.1371/journal.pone.0154075

**Published:** 2016-04-26

**Authors:** Jenna B. Gillen, Brian J. Martin, Martin J. MacInnis, Lauren E. Skelly, Mark A. Tarnopolsky, Martin J. Gibala

**Affiliations:** 1 Department of Kinesiology, McMaster University, Hamilton, ON, Canada; 2 Department of Pediatrics and Medicine, McMaster University, Hamilton, ON, Canada; Norwegian University of Science and Technology, NORWAY

## Abstract

**Aims:**

We investigated whether sprint interval training (SIT) was a time-efficient exercise strategy to improve insulin sensitivity and other indices of cardiometabolic health to the same extent as traditional moderate-intensity continuous training (MICT). SIT involved 1 minute of intense exercise within a 10-minute time commitment, whereas MICT involved 50 minutes of continuous exercise per session.

**Methods:**

Sedentary men (27±8y; BMI = 26±6kg/m^2^) performed three weekly sessions of SIT (n = 9) or MICT (n = 10) for 12 weeks or served as non-training controls (n = 6). SIT involved 3x20-second ‘all-out’ cycle sprints (~500W) interspersed with 2 minutes of cycling at 50W, whereas MICT involved 45 minutes of continuous cycling at ~70% maximal heart rate (~110W). Both protocols involved a 2-minute warm-up and 3-minute cool-down at 50W.

**Results:**

Peak oxygen uptake increased after training by 19% in both groups (SIT: 32±7 to 38±8; MICT: 34±6 to 40±8ml/kg/min; p<0.001 for both). Insulin sensitivity index (CS_I_), determined by intravenous glucose tolerance tests performed before and 72 hours after training, increased similarly after SIT (4.9±2.5 to 7.5±4.7, p = 0.002) and MICT (5.0±3.3 to 6.7±5.0 x 10^−4^ min^-1^ [μU/mL]^-1^, p = 0.013) (p<0.05). Skeletal muscle mitochondrial content also increased similarly after SIT and MICT, as primarily reflected by the maximal activity of citrate synthase (CS; P<0.001). The corresponding changes in the control group were small for VO_2_peak (p = 0.99), CS_I_ (p = 0.63) and CS (p = 0.97).

**Conclusions:**

Twelve weeks of brief intense interval exercise improved indices of cardiometabolic health to the same extent as traditional endurance training in sedentary men, despite a five-fold lower exercise volume and time commitment.

## Introduction

Regular exercise training is well accepted as an effective therapeutic intervention for the prevention and treatment of many chronic diseases, including type 2 diabetes [1,2]. Endurance exercise training enhances cardiorespiratory fitness [3], induces skeletal muscle remodelling towards a more oxidative phenotype [4] and promotes favourable changes in insulin sensitivity [1]. These well-established health benefits provide support for current physical activity guidelines that recommend 150 minutes of moderate-intensity or 75 minutes of vigorous-intensity aerobic physical activity per week [5–7]. Despite the association between low amounts of physical activity and increased risk of many chronic diseases, the prevalence of physical inactivity is higher than that of all other modifiable risk factors [2]. The reasons for not engaging in regular physical activity are numerous and complex, but “lack of time” remains one of the most commonly cited barriers [8]. Therefore, developing more time-efficient, yet equally effective exercise strategies are urgently needed.

In contrast to traditional endurance training, sprint interval training (SIT) is characterized by brief intermittent bursts of relatively intense exercise separated by periods of low-intensity exercise for recovery [9]. A commonly studied SIT model is repeated Wingate Tests; typically, four to six 30-second “all-out” cycling efforts are performed per session, interspersed by 4 minutes of recovery. Studies that have directly compared several weeks of Wingate-based SIT to moderate-intensity continuous training (MICT) have reported similar improvements in cardiorespiratory fitness [10], skeletal muscle oxidative capacity [10,11] and insulin sensitivity based on oral glucose tolerance tests [12,13].

Given that Wingate-based SIT involves ~20–30 minutes per session, not including warm-up or cool-down, the purported “time efficiency” of this type of training has been questioned [14]. Recent studies have shown that very brief SIT protocols involving ≤10 min per session elicit adaptations similar to longer SIT protocols and MICT [15–17]. For example, a cycling protocol involving three, 20-second ‘all-out’ sprints, within a 10 minute training session including warm-up and cool-down, improved cardiorespiratory fitness and reduced 24-hour average blood glucose concentration in overweight adults when performed three times per week for 6 weeks [17]. No study has directly compared this type of very brief SIT protocol to traditional endurance training, nor measured changes in glycemic control using a robust measure of insulin sensitivity.

The purpose of the present study was to compare the effects of 12 weeks of SIT or MICT on insulin sensitivity and other indices of cardiometabolic health including cardiorespiratory fitness and skeletal muscle mitochondrial content in sedentary men. The two protocols differed markedly with respect to total exercise volume and time commitment: SIT involved 1 minute of intense intermittent exercise within a 10-minute session, whereas MICT consisted of 50 minutes of moderate-intensity continuous exercise. We hypothesized that compared to a non-training control group (CTL), SIT and MICT would similarly increase insulin sensitivity based on the intravenous glucose tolerance test method, cardiorespiratory fitness as determined by a peak oxygen uptake (VO_2peak_) test and mitochondrial content as reflected by the maximal activity of citrate synthase.

## Methods

### Subjects and Ethics Approval

Twenty-seven sedentary men took part in the study. Participants were generally deemed inactive based on an International Physical Activity Questionnaire score of less than 600 MET-minutes per week. Participants were matched for age, BMI and VO_2_peak, and assigned to SIT, MICT or CTL. One subject in each of the two training groups dropped out for reasons unrelated to the study, resulting in n = 9, 10 and 6 in SIT, MICT and CTL, respectively (Table 1). The experimental protocol was approved by the Hamilton Integrated Research Ethics Board. All participants provided written informed consent.

**Table 1:** Subject Characteristics.

VARIABLE	MICT (10)	SIT (9)	CTL (6)
**Age (y)**	28 ± 9	27 ± 7	26 ± 8
**Height (cm)**	176 ± 10	177 ± 11	176 ± 5
**Weight (kg)**	84 ± 20	84 ± 23	78 ± 25
**Body Mass Index (kg/m**^**2**^**)**	26 ± 6	27 ± 5	25 ± 7
**VO**_**2**_**peak (ml/kg/min)**	33 ± 6	32 ± 7	32 ± 7
**VO**_**2**_**peak (L/min)**	2.7 ± 0.5	2.6 ± 0.8	2.5 ± 0.7
**Maximal Workload (W)**	248 ± 30	243 ± 68	219 ± 60

Values are means ± S.D. VO_2_peak, maximal oxygen uptake. No differences were observed between groups at baseline for any variable.

### Experimental Protocol

#### Baseline testing and exercise familiarization

Participants performed an incremental VO_2_peak test on an electronically-braked cycle ergometer (Lode Excalibur Sport V 2.0, The Netherlands), as described previously [17]. Briefly, following a 1-minute warm-up at 50 W, the resistance was increased by 1 W every 2 seconds until exhaustion or when pedal cadence fell below 50 rpm. For all tests an RER >1.1 was achieved. Oxygen consumption and carbon dioxide production data were acquired through a metabolic cart with an online gas collection system (Moxus, AEI Technologies, PA), and VO_2_peak was defined as the highest average oxygen consumption over 30 seconds.

Approximately 5 days later and following a 10-hour overnight fast, participants underwent a body composition test and a 50-minute intravenous glucose tolerance test (IVGTT). Participants consumed a standardized meal the evening before the visit consisting of 561±99kcal (47±2% carbohydrate, 31±3% fat and 22±4% protein). Fat mass was determined through air-displacement plethysmography (BodPod®,COSMED). Subsequently, a trained nurse inserted two indwelling catheters into forearm veins (one in each arm). A fasting blood sample (12ml) was obtained from the “sampling arm”, and glucose (0.5g/kg up to 35g) was manually delivered to the contralateral “infusion arm” over 3 minutes. A 38±2% glucose solution (Hospira LifeCare) was used in a total volume of 90ml. Blood samples (8ml) were obtained from the “sampling arm” every 10 minutes for 50 minutes post-infusion. Plasma and serum were separated by centrifugation and stored at -80°C.

Approximately 2 days later, a resting muscle biopsy from the *vastus lateralis* (~100mg) was obtained using the Bergström needle adapted with suction, as described previously [18]. Briefly, a single muscle sample (~100 mg) was obtained from the *vastus lateralis* under local anesthesia (1% lidocaine) using a Bergström needle adapted with suction. Samples were sectioned into several pieces, snap frozen in liquid nitrogen and stored at -80°C for later analysis.

At least 5 days following the muscle biopsy, exercise familiarization took place. Participants in SIT performed two 20-second ‘all-out’ sprints on an electronically-braked ergometer (Veletron, RacerMate, USA). Participants in MICT were fitted with a heart rate (HR) monitor (Polar A3, Lake Success, USA) and cycled on an ergometer (Kettler, Ergo Race I, Germany) for ~20 minutes to determine the workload that elicited 64–76% of maximal heart rate (HR_max_). The target HR for MICT was based on the classification for “moderate-intensity” put forth by the American College of Sports Medicine [6].

#### 12-week training intervention

Training involved a lead-in phase, in which one session was completed in week 1, and two sessions in week 2. Exercise was performed three times per week thereafter, with the exception of week 7 where two sessions were replaced with a “mid-training assessment” for VO_2_peak and arterial ultrasound imaging (a collaborative measure not reported in the present manuscript). During training, a HR monitor recorded HR every 5 seconds, from which average HR during each session was determined. The SIT protocol consisted of 3x20-second ‘all-out’ cycling efforts against 0.05kg/kg body mass, separated by 2 minutes of low-intensity cycling (50W). The MICT protocol consisted of 45 minutes of continuous cycling at ~70% HR_max_. A 2-minute warm-up and 3-minute cool-down at 50W were included, resulting in 10- and 50-minute sessions for SIT and MICT, respectively. To accommodate progression, training loads were adjusted to maintain the desired relative exercise intensity. Ratings of perceived exertion (RPE; Borg 6–20 scale) were recorded at the end of each sprint (SIT) or at 15, 30 and 45 minutes of exercise (MICT), on the 1^st^, 15^th^ and 30^th^ sessions. Participants in CTL did not report to the laboratory during the 12-week intervention, with the exception of week 7 for mid-assessment.

#### Post-testing

Participants repeated the body composition test and IVGTT 72 hours after training cessation. A resting muscle biopsy was obtained 24 hours later, or 96 hours post-training. A VO_2_peak test was performed approximately 4 days after the biopsy and 1 week after training. All procedures were identical to baseline testing.

#### Glucose and Insulin Assays

Plasma glucose was analyzed (Pointe Scientific, USA), and serum insulin was measured with ELISA (ALPCO Immunoassays, USA). The insulin sensitivity index (CS_I_) from the 50-minute IVGTT was calculated as proposed by Tura et al. [19]. CS_I_ is highly correlated with the Minimal Model insulin sensitivity index (S_I_) obtained from a 3-hour IVGTT, as well as the glucose infusion rate during a hyperinsulinemic-euglycemic clamp [19]. This method has also been used to assess insulin sensitivity in response to acute exercise [20,21], and has greater reproducibility than the Matsuda composite index (M_ISI_) derived from an OGTT [20]. Briefly, CS_I_ was calculated as follows:

$$\text{CSI}=\;\alpha \;\left\lceil \frac{\text{KG}}{\left(\frac{\Delta \text{AUCINS}}{\text{T}}\right)}\right\rceil$$

where α is a scaling factor (0.604), K_G_ is the glucose disappearance rate (mmol/L; calculated as the slope of log [glucose]), ΔAUC_INS_ is the insulin area under the curve above basal (uIU/ml) and T is the time between 10 and 50 minutes (40 minutes) from which K_G_ and ΔAUC_INS_ were calculated [19].

Delta glucose and insulin area under the curve (AUC) from 0–50 minutes were also calculated, and fasting insulin resistance was determined using HOMA-IR [22].

#### Muscle Analysis

For enzyme activity, one piece of muscle (~25mg) was homogenized as described previously [17]. The maximal activities of citrate synthase (CS) and 3-β-hydroxyacyl CoA dehydrogenase (β-HAD) were determined using established techniques [23]. Samples were run in duplicate and the intra-assay coefficient of variation for CS and β-HAD were 3.0 and 6.5%, respectively. Protein concentration was determined (BCA Protein Assay, Pierce, USA) and enzyme activity is expressed as mmol/kg protein/h.

For western blotting, a piece of muscle (~30mg) was homogenized in RIPA buffer as previously described [17] and western blot analysis was conducted using established techniques [17,24]. ImageJ software was used to quantify the optical density of protein bands. α-tubulin (Cell Signaling Technology, #2125), which did not change following training (p = 0.85), was used as a loading control. The following primary antibodies from Mitosciences were used: NDUFA9 (MS111), CII-70 kDa subunit (MS204), CIII-Core protein 2 (MS304), CIV subunit IV (MS408), ATP synthase α-subunit (MS507) and GLUT4 (Millipore, AB1345).

#### Statistics

Baseline characteristics (Table 1) were analyzed using a one-way (group) analysis of variance (ANOVA). Muscle, blood, VO_2_peak and body composition data were analyzed using a two-way ANOVA with the between factor, group (levels: SIT, MICT, CTL) and the within factor, time (levels: pre- and post-training for all variables except for VO_2_peak which also included a mid-training time point). Significant group x time interactions (p<0.05) were analyzed using a Tukey’s honestly significant difference post hoc test. All analyses were conducted using SPSS software, and significance was set at p<0.05. Data are presented as means±S.D. for n = 10 (MICT), n = 9 (SIT) and n = 6 (CTL). Due to difficulties during data collection, we report n = 9 (MICT) and n = 5 (CTL) for body composition data and n = 8 (SIT) for blood analyses.

## Results

### Descriptive characteristics of training

A total of 31±1 and 32±2 sessions were completed in SIT and MICT, respectively. Mean HR, averaged over all training sessions, was 79±4% and 71±5% of HR_max_ for SIT and MICT, respectively. Mean RPE, measured during the 1^st^, 15^th^ and 30^th^ exercise sessions, was 16±1 for SIT and 13±1 for MICT. Mean total work was ~60 and ~310kJ per session for SIT and MICT, respectively. Body mass remained similar over the course of the study in all groups. Percent body fat decreased after SIT (p = 0.011) and MICT (p = 0.011) whereas there was little change in CON (p = 0.12) (Table 2). Change scores with 95% confidence intervals are available in a supplemental file for all training-induced outcomes summarized in Table 2 (S1 Fig).

**Table 2:** Descriptive Characteristics and Markers of Glycemic Control.

VARIABLE	MICT (10)		SIT (9)		CTL (6)		STATISTICS		
	PRE	POST	PRE	POST	PRE	POST	Time	Group	T x G
**Weight (kg)**	84 ± 20	82 ± 20	84 ± 23	83 ± 22	78 ± 25	78 ± 23	0.111	0.875	0.364
**BMI (kg/m**^**2**^**)**	26 ± 6	26 ± 6	27 ± 5	26 ± 5	25 ± 7	25 ± 7	0.125	0.869	0.334
**Percent Fat (%)**	27 ± 10	25 ± 10*	30 ± 7	28 ± 8*	24 ± 6	25 ± 6	0.098	0.546	0.012
**VO**_**2**_**peak (L/min)**	2.7 ± 0.5	3.2 ± 0.5*	2.6 ± 0.8	3.0 ± 0.7*	2.5 ± 0.7	2.5 ± 0.7	<0.0001	0.338	<0.0001
**Max Workload (W)**	248 ± 30	271 ± 33*	243 ± 68	275 ± 50*	219 ± 60	213 ± 52	<0.0001	0.141	<0.0001
**CS**_**I**_	5.0 ± 3.3	6.7 ± 5.0*	4.9 ± 2.5	7.5 ± 4.7*	7.4 ± 5.8	7.0 ± 4.9	0.006	0.841	0.039
**K**_**G**_ **(%/min)**	2.0 ± 0.9	2.1 ± 0.7	2.1 ± 0.9	2.4 ± 0.8	2.1 ± 0.6	2.1 ± 0.7	0.119	0.822	0.576
**ΔAUC**_**INS (10–50 min)**_ **(uIU/ml)**	1171 ± 591	1007 ± 545	1231 ± 705	1149 ± 844	1095 ± 843	1158 ± 908	0.338	0.956	0.353
**ΔInsulin AUC (uIU/ml)**	1423 ± 712	1223 ± 647	1515 ± 917	1454 ± 1065	1317 ± 946	1425 ± 1035	0.463	0.922	0.209
**ΔGlucose AUC (mmol/L)**	321 ± 144	257 ± 103*	303 ± 92	225 ± 75*	201 ± 52	222 ± 54	0.001	0.235	0.004
**FPG (mmol/L)**	5.3 ± 0.8	5.7 ± 0.9	5.0 ± 1.2	5.4 ± 0.8	5.5 ± 1.6	5.4 ± 0.8	0.164	0.841	0.284
**FPI (uIU/mL)**	10.1 ± 6.0	8.4 ± 6.9	9.5 ± 5.3	7.8 ± 4.1	7.5 ± 6.4	10.8 ±13.2	0.854	0.992	0.135
**HOMA-IR**	2.4 ± 1.6	2.3 ± 2.1	2.1 ± 1.3	1.9 ± 1.0	2.0 ± 2.3	2.7 ± 3.7	0.465	0.92	0.348
**GLUT4 Protein Content**	1.0 ± 0.6	1.5 ± 0.6*	1.0 ± 0.6	1.6 ± 0.6*	1.0 ± 0.4	0.9 ± 0.4	0.003	0.403	0.021

Values are means ± S.D. **VO**_**2**_**peak**, maximal oxygen uptake; **CS**_**I**_, insulin sensitivity index from IVGTT; **K**_**G**_, glucose rate of disappearance during 10–50 min of IVGTT; **ΔAUC**_**INS**_, insulin area under the curve from 10–50 min of IVGTT; **ΔInsulin AUC**, insulin area under the curve from 0–50 min of IVGTT; **ΔGlucose AUC**, glucose area under the curve from 0–50 min of IVGTT; **FPG**, fasting plasma glucose; **FPI**, fasting plasma insulin.

*Significantly different vs. pre-training (p<0.05), as determined by post-hoc analyses following a significant Time x Group interaction (T x G).

### Cardiorespiratory fitness

**Fig 1:**
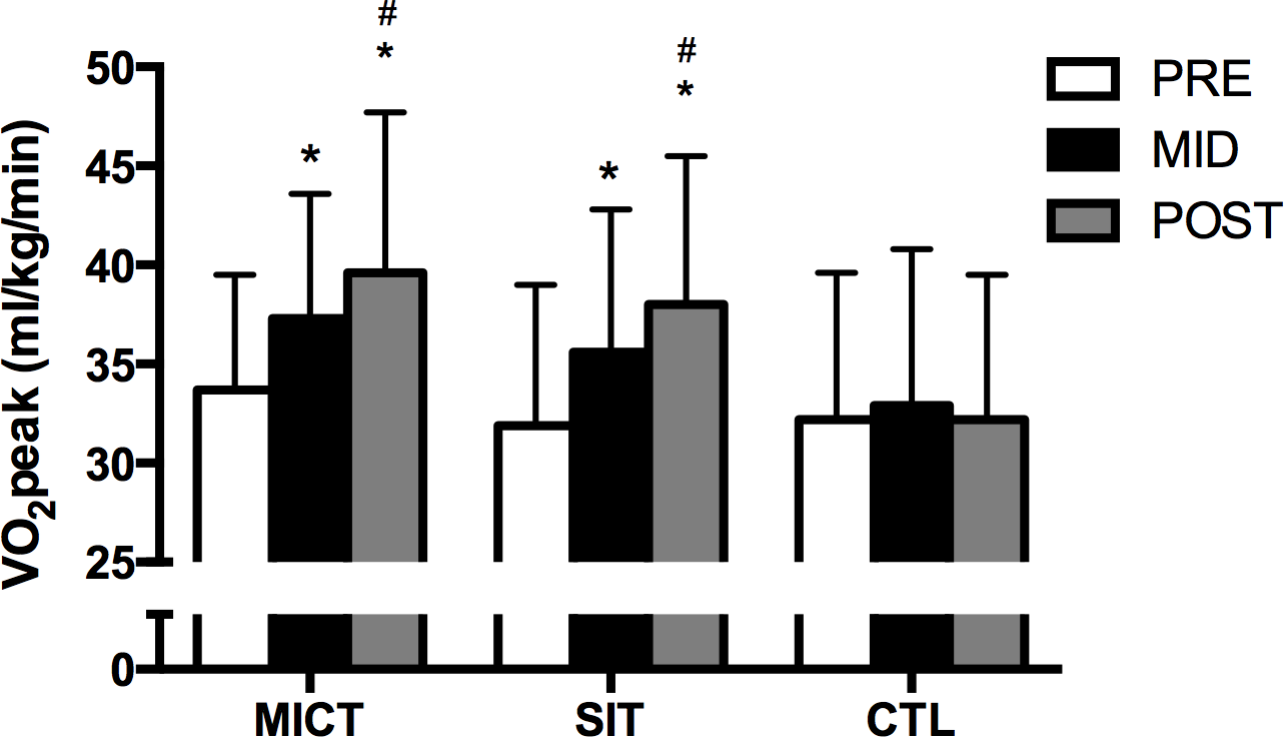
Effect of SIT and MICT on VO_2_peak. Measured at baseline (PRE), 6 weeks (MID), and 12 weeks (POST) in MICT, SIT and CTL. Values are means ± S.D. * p<0.05, vs. same group at PRE; # p<0.05, vs. same group at MID.

VO_2_peak increased compared to pre-training by ~12% after 6 weeks of both SIT and MICT (p<0.001 for both). VO_2_peak increased further after 12 weeks compared to 6 weeks (p = 0.007 and p = 0.005 for SIT and MICT, respectively), resulting in a 19% overall increase versus pre-training (p<0.001 for both; Fig 1). The CTL group showed only small changes from baseline when measured at both 6 (p = 0.43) and 12 (p = 0.99) weeks.

### Indices of glycemic control

**Fig 2:**
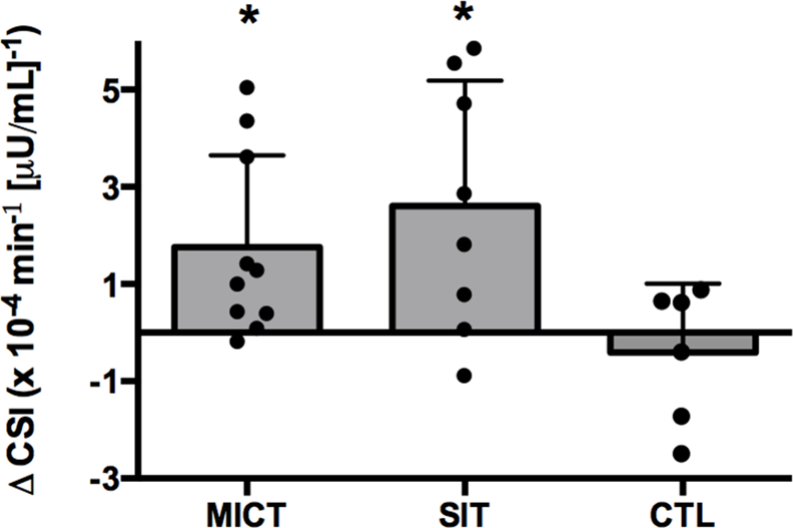
Effect of SIT and MICT on insulin sensitivity. The change in insulin sensitivity (CS_I_) over the 12-week intervention, measured from a 50-minute IVGTT in MICT, SIT and CTL. Closed circles denote individual responses. Values are means ± S.D. * p<0.05, PRE vs. POST.

CS_I_ increased by 53 and 34% after 12 weeks of SIT (p = 0.002) and MICT (p = 0.013; Fig 2) whereas the change was small in CON (p = 0.64). Glucose AUC during the 50-minute IVGTT was reduced to a greater extent after SIT (p<0.001) and MICT (p = 0.001) compared to CON (p = 0.32). These data and other fasting indices of glycemic control are summarized in Table 2.

### Skeletal muscle adaptations

**Fig 3:**
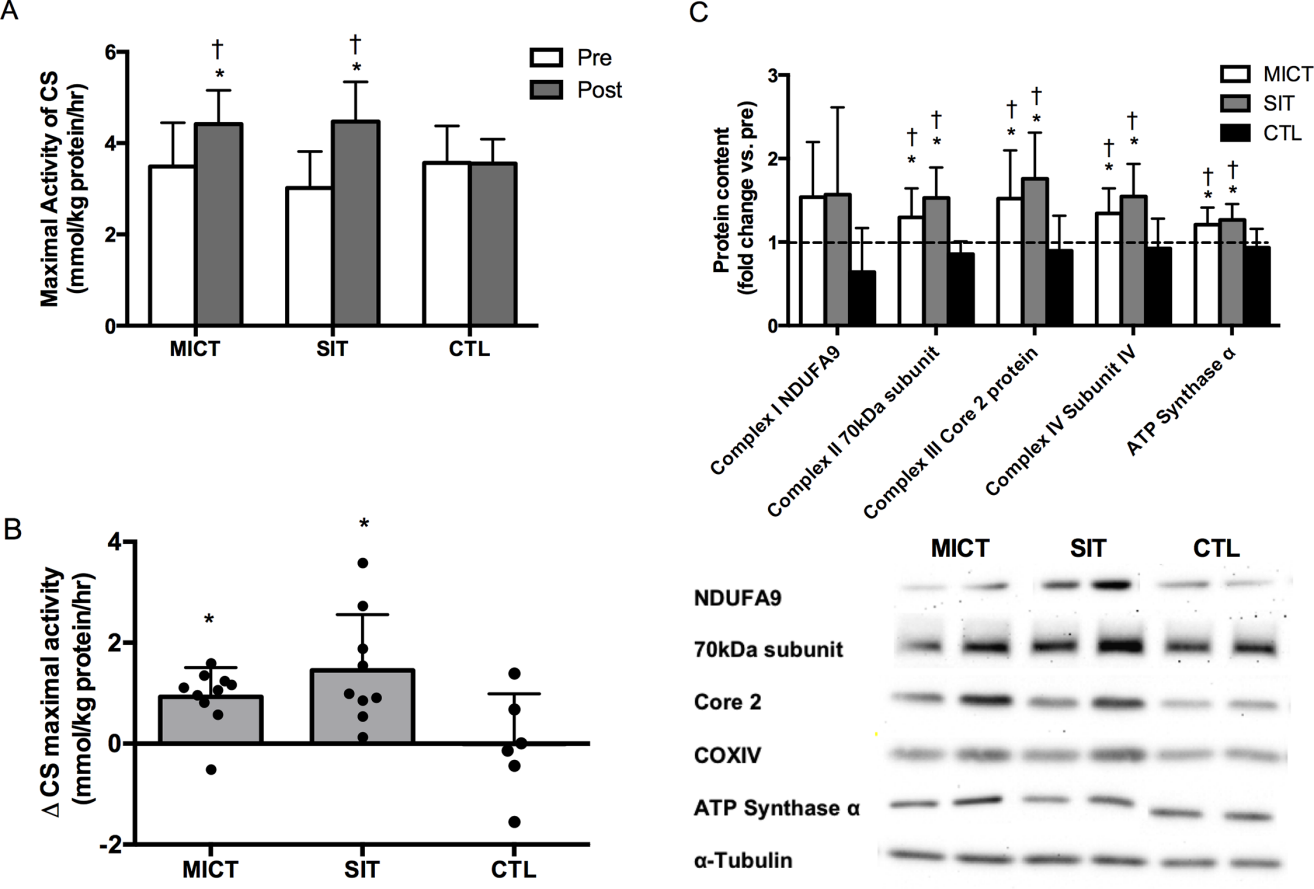
Effect of SIT and MICT on skeletal muscle mitochondrial content. Measured in muscle biopsy samples obtained from the vastus lateralis before (PRE) and 96 h after (POST) the 12-week intervention in MICT, SIT and CTL. Maximal activity of citrate synthase (A), individual changes in maximal activity of citrate synthase (B) and protein content of various subunits from complexes in the electron transport chain (C). Representative western blots are shown. Values are means ± S.D. * p<0.05, vs. same group at PRE; † p<0.05, vs. CTL at POST.

The maximal activity of CS increased by 48 and 27% after 12 weeks of SIT (p<0.0001) and MICT (p = 0.004), respectively, and was higher than CTL post-training (p = 0.03 for both; Fig 3). Training also increased the protein of Complex II-70kDa (SIT: p<0.001; MICT: p = 0.02) Complex III-Core protein 2 (SIT: p<0.001; MICT: p = 0.003), COX subunit IV (SIT: p<0.001; MICT: p = 0.001) and ATP Synthase α-subunit (SIT: p = 0.001; MICT: p = 0.004), all of which were higher than CTL post-training (p<0.05; Fig 3). The absolute increase in β-HAD maximal activity was 28% and 17% in SIT and MICT, respectively, compared to a change of -2% in CTL, but a time by group interaction was not observed (p = 0.16). GLUT4 protein content increased by ~50% after SIT (p = 0.001) and MICT (p-0.002), whereas there was little change in CTL (p = 0.50; Table 2).

## Discussion

The major novel finding from the present study was that 12 weeks of SIT in previously inactive men improved insulin sensitivity, cardiorespiratory fitness, and skeletal muscle mitochondrial content to the same extent as MICT, despite a five-fold lower exercise volume and training time commitment. SIT involved 1 minute of intense intermittent exercise, within a time commitment of 10 minutes per session, whereas MICT consisted of 50 minutes of continuous exercise at a moderate pace. A few previous studies have reported similar improvements in skeletal muscle remodeling and markers of health status after SIT and MICT lasting up to 6 weeks [10,11,13]. The present work was intended to be more ambitious in scope as compared to previous studies that compared SIT versus MICT. Specifically, it involved a SIT protocol that required less total time commitment than in previous studies (i.e., 10 vs ~25 min), a training program that was twice as long (i.e., 12 vs 6 wk), a more robust measure of insulin sensitivity (i.e., an IVGTT vs OGTT and fasting blood measures of insulin sensitivity), and inclusion of a non-training control group.

### Cardiorespiratory fitness

Low cardiorespiratory fitness is a strong independent risk factor for cardiovascular disease and all-cause mortality [25,26]. It has been known for decades that interval training involving brief hard efforts is a potent stimulus to improve cardiorespiratory fitness [27,28]. Recent studies have shown that protocols involving as little as one minute of sprint interval training per session can be very effective in this regard [15–17]. Prior to the present work, no study had directly compared this type of SIT protocol with traditional endurance training as reflected in public health guidelines. We found a strikingly similar 19% improvement in VO_2_peak after 12 weeks of SIT and MICT, which compares favorably with the typical change reported after several months of traditional endurance training [29,30].

Exercise intensity is generally regarded to be the more critical factor in the trainability of VO_2_peak, with higher intensity exercise conferring larger improvements in cardiorespiratory fitness when exercise is matched for total energy expenditure [31–34]. This message was recently reinforced by Ross et al. [35], who found that low-intensity exercise (50% VO_2_peak) performed for about 150 minutes per week, over 24 weeks, may not be sufficient to improve cardiorespiratory fitness for a substantive proportion of adults. In contrast, the present data show it is possible for previously sedentary individuals to markedly improve VO_2_peak by performing a total of 3 minutes per week of short intense bursts of exercise, within a 30-minute time commitment, over 12 weeks. The absolute change in relative VO_2_peak was ~6 ml/kg/min in both SIT and MICT, which corresponds to ~1.7 metabolic equivalents (METs). These findings are noteworthy given that a 1-MET increase in cardiorespiratory is comparable to a 7 cm decrease in waist circumference, a 5 mmHg lowering of systolic blood pressure or a 1 mmol/L reduction in fasting plasma glucose, in terms of relative risk reduction in all-cause and cardiovascular disease mortality [25]. Unfit individuals also have twice the risk of death regardless of BMI, while fit and overweight/obese adults have similar mortality risk as their normal weight counterparts [26].

The precise mechanisms responsible for the improved cardiorespiratory fitness observed after SIT and MICT in the present study are unknown. The increase in VO_2_peak after traditional endurance training is generally attributed to an enhanced cardiac output owing to a greater stroke volume, although numerous factors may contribute to this adaptive response [3]. A limited number of studies have assessed cardiovascular adaptations to SIT and these have yielded equivocal results, likely due in part to differences in experimental design as well as the specific analytical procedures employed [36,37]. Increases in resting stroke volume have been observed after 7 weeks of SIT using cardiac MRI [38], as has stroke volume during submaximal exercise based on the CO_2_-rebreathing technique following 4 weeks of SIT [36]. Conversely, MacPherson et al. [37] reported no changes in maximal cardiac output based on the acetylene non-rebreathing technique after 6 weeks of run-based SIT. It has also been suggested that peripheral factors that enhance oxygen extraction may contribute to SIT-induced improvements in VO_2_peak, at least over the short-term [39]. Additional studies are warranted to clarify both the time course and precise mechanisms responsible for the improved cardiorespiratory fitness after SIT compared to MICT.

### Insulin sensitivity

Perhaps the most striking and novel finding form the present work was the similar increase in insulin sensitivity after SIT and MICT. It has previously been shown that SIT improves indices of glycemic control, as determined by the hyperinsulinemic-euglycemic clamp method [40], continuous glucose monitoring [17] and oral glucose tolerance tests [13,41]. In the present investigation, we employed a 50-minute IVGTT (CS_I_), which was recently validated by Tura and colleagues as a robust marker of insulin sensitivity [19]. The technique was shown to be highly correlated with the gold standard glucose infusion rate obtained during a hyperinsulinemic-euglycemic clamp [19], and have greater reproducibility than M_ISI_ derived from OGTTs [20].

Houmard et al. [42], using the IVGTT method, previously reported that a continuous training protocol involving 170 minutes of exercise per week improved insulin sensitivity to a greater extent than 115 minutes per week, regardless of exercise intensity and volume. Several recent reports, however, suggest that when exercise is matched for total volume or energy expenditure, higher-intensity exercise training confers larger improvements in insulin sensitivity in individuals with obesity [43], metabolic syndrome [33] and type 2 diabetes [31,34]. Our findings support this general concept and demonstrate that a surprisingly small amount of high-intensity exercise can be as effective as a large volume of moderate-intensity continuous exercise for improving insulin sensitivity.

The potential mechanisms that mediate exercise training-induced increases in whole-body insulin sensitivity are obviously complex [1]. With respect to potential changes in skeletal muscle that might in part explain the improved insulin sensitivity, we found similar increases in GLUT4 protein content after the two training protocols despite large differences in exercise volume. SIT and MICT have also been shown to similarly increase skeletal muscle microvascular density [44], which is associated with improved glucose transport and insulin sensitivity [44,45]. It is also possible that the improvement in mitochondrial content [1] or an increased capacity for intramuscular triglyceride utilization [12] could be involved.

### Mitochondrial content

Reduced skeletal muscle mitochondrial content is associated with aberrant lipid handling, poor insulin sensitivity and an impaired metabolic health profile [46]. Physical activity increases mitochondrial content and insulin sensitivity, but it remains unclear whether these effects are directly linked [1,46]. The maximal activity of citrate synthase is a commonly measured marker that is strongly associated with mitochondrial content in human skeletal muscle [47]. A novel finding from the present work was the similar increase in citrate synthase maximal activity after 12 weeks of SIT and MICT, despite the large difference in total exercise volume. We also observed similar increases in the protein content of various subunits from complexes in the electron transport chain, highlighting similar mitochondrial adaptation in both groups. We did not examine the time course of skeletal muscle remodeling, but the mean increase in CS maximal activity after training was similar to the 30–40% increase that we previously observed after 2 [48] and 6 weeks [10,17] of both SIT and MICT. Consistent with the recent observations by Egan et al. [49], who examined the time course of increased mitochondrial content in response to 14 sessions of endurance training, these data seem to imply that much of the increase in mitochondrial content occurs relatively early in response to training. Given the much lower exercise volume involved with SIT, these data also seemingly suggest that training intensity, rather than volume, may be the more critical determinant of the improvement in mitochondrial content. As recently reviewed by Bishop et al. [50], a surprisingly small number of studies have investigated the impact of varying training intensity and volume on changes in mitochondrial function and content in human skeletal muscle, and additional work in this regard is warranted.

### Efficacy versus Effectiveness

While the present study and work by others highlights the efficacy of SIT for improving indices of cardiometabolic health, the potential effectiveness of interval training in its various forms and likely impact on public health remains contentious [51]. Research in the field of exercise behavior has demonstrated a negative relationship between exercise intensity and affect, particularly in less trained individuals, which suggests people are less likely to adhere to a program of vigorous exercise since it is deemed aversive [52,53]. However, in a recent study by Jung et al [54], subjects reported greater enjoyment of, and a preference to engage in, a high-intensity intermittent exercise protocol as compared to continuous moderate- or vigorous-intensity exercise. The interval protocol involved 20 minutes of alternating 60-second periods of exercise at 100% and 20% Wpeak, whereas the continuous protocols involved exercise at 40% Wpeak for 40 minutes or 80% Wpeak for 20 minutes. Other work by the same authors showed that adherence to a 4-week high-intensity interval training program, assessed by self-report in free-living conditions, was greater than for moderate-intensity continuous exercise in people with prediabetes [55]. These findings highlight the potential utility of intense interval training as an alternative exercise strategy that could bolster exercise adherence, but longer and more comprehensive studies are warranted in this regard.

## Conclusion

In summary, we report that a SIT protocol involving 3 minutes of intense intermittent exercise per week, within a total time commitment of 30 minutes, is as effective as 150 minutes per week of moderate-intensity continuous training for increasing insulin sensitivity, cardiorespiratory fitness and skeletal muscle mitochondrial content in previously inactive men. This investigation represents the longest comparison of SIT and MICT to date and demonstrates the efficacy of brief, intense exercise to improve indices of cardiometabolic health. While SIT is clearly a potent stimulus to elicit physiological adaptations, this type of exercise requires a very high level of motivation and is clearly not suited for everyone. Future studies should examine the potential for interval training protocols that involve relatively intense but not “all out” efforts to elicit changes like that shown in the present study. Considering that a large number of individuals do not meet the current physical activity recommendations [56,57], there is value in exploring the potential benefits of exercise strategies that involve reduced time commitment. Large-scale randomized clinical trials are needed to advance the field.

## Supporting Information

S1 Fig Descriptive Characteristics and Markers of Glycemic Control. Values represent change scores (95% confidence intervals). **VO**_**2**_**peak**, maximal oxygen uptake; **CS**_**I**_, insulin sensitivity index from IVGTT; **K**_**G**_, glucose rate of disappearance during 10–50 min of IVGTT; **ΔAUC**_**INS**_, insulin area under the curve from 10–50 min of IVGTT; **ΔInsulin AUC**, insulin area under the curve from 0–50 min of IVGTT; **ΔGlucose AUC**, glucose area under the curve from 0–50 min of IVGTT; **FPG**, fasting plasma glucose; **FPI**, fasting plasma insulin.
(PDF)
