# Supplementary material for: Twelve Weeks of Sprint Interval Training Improves Indices of Cardiometabolic Health Similar to Traditional Endurance Training despite a Five-Fold Lower Exercise Volume and Time Commitment
Source: PLoS One. 2016 Apr 26;11(4):e0154075. doi: 10.1371/journal.pone.0154075 (PMC4846072; doi:10.1371/journal.pone.0154075)
Supplement: S1 Fig — Values represent change scores (95% confidence intervals). VO2peak, maximal oxygen uptake; CSI, insulin sensitivity index from IVGTT; KG, glucose rate of disappearance during 10–50 min of IVGTT; ΔAUCINS, insulin area under the curve from 10–50 min of IVGTT; ΔInsulin AUC, insulin area under the curve from 0–50 min of IVGTT; ΔGlucose AUC, glucose area under the curve from 0–50 min of IVGTT; FPG, fasting plasma glucose; FPI, fasting plasma insulin. (PDF) [file pone.0154075.s001.pdf]

VARIABLE	Mean Difference (95% CI)		
	MICT	SIT	CTL
Weight (kg)	-1.4 (-2.7, -0.1)	-1.8 (-4.2, 0.6)	0.2 (-2.5, 2.9)
BMI (kg/m^2)	-0.5 (-0.9, -0.1)	-0.5 (-1.3, 0.2)	0.1 (-0.8, 1.0)
Percent Fat (%)	-1.6 (-2.5, -0.6)	-2.1 (-3.9, -0.3)	1.2 (-0.5, 2.8)
VO2peak (ml/kg/min)	6.0 (3.7, 8.3)	5.9 (4.0, 7.8)	-0.02 (-0.9, 0.9)
VO_2peak (L/min)	0.4 (0.2, 0.7)	0.4 (0.2, 0.5)	-0.02 (-0.2, 0.1)
Max Workload (W)	23 (13, 33)	30 (12, 48)	-6 (-23, 12)
CS_I	1.8 (0.4, 3.1)	2.6 (0.5, 4.8)	-0.4 (-1.9, 1.1)
K_G (%/min)	0.12 (-0.28, 0.53)	0.33 (-0.13, 0.78)	0.07 (-0.20, 0.34)
$\Delta AUC_{INS\ (10-50\ min)}$ (uIU/ml)	-163 (-349, 22)	-81 (-417, 255)	109 (-83, 300)
ΔInsulin AUC (uIU/ml)	-200 (-430, 30)	-61 (-397, 274)	109 (-83, 300)
ΔGlucose AUC (mmol/L)	-64 (-105, -22)	-78 (-124, -32)	22 (-15, 58)
FPG (mmol/L)	-0.1 (-1.8, 1.6)	0.4 (-0.6, 1.3)	-0.2 (-1.1, 0.9)
FPI (uIU/mL)	-2.6 (-6.6, 1.5)	-1.6 (-3.9, 0.6)	3.3 (-4.7, 11.4)
HOMA-IR	-0.4 (-1.8, 1.0)	-0.2 (-0.7, -0.3)	0.7 (-0.8, 2.2)
